# Ultrasound‐guided cyst aspiration for management of acute adnexal torsion

**DOI:** 10.1002/uog.29225

**Published:** 2025-04-26

**Authors:** L. Berg, N. Eagles, S. Kastora, J. Farren, J. Naftalin, D. Jurkovic

**Affiliations:** ^1^ EGA Institute for Women's Health, Faculty of Population Health Sciences University College London London UK

**Keywords:** aspiration, cyst, ovarian torsion, treatment, ultrasound

## Abstract

**Objective:**

Ultrasound‐guided cyst aspiration is a potential treatment for acute adnexal torsion that can be performed in the outpatient setting, offering an alternative to emergency laparoscopic surgery. The objective of this study was to describe our initial experience with aspiration of acutely torted adnexal cysts.

**Methods:**

This was a retrospective single‐center study, in which we identified all patients who underwent ultrasound‐guided cyst aspiration in our center between February 2008 and September 2023. Indications for the procedure were ultrasound diagnosis of adnexal torsion due to an ovarian cyst and clinical symptoms of acute adnexal torsion. Success was defined as the avoidance of emergency surgery for adnexal torsion related to the same cyst. We extracted clinical data, ultrasound scan findings, procedural details and clinical outcomes from our clinical database.

**Results:**

Overall, 46 patients underwent ultrasound‐guided cyst aspiration for the management of adnexal torsion, 24 (52%) of whom were pregnant. At ultrasound examination, all 46 cysts had unilocular morphology, and in 96% (44/46) of cases, the cyst content was anechoic. Cyst aspiration was performed transabdominally in 30 (65%) patients and transvaginally in 16 (35%) patients. A total of 39 (85%) patients experienced complete resolution of symptoms immediately after ultrasound‐guided cyst aspiration. One procedure was abandoned owing to patient discomfort and six (13%) patients reported non‐resolution or short‐term recurrence of symptoms, necessitating emergency laparoscopy in seven patients. No complications occurred as a result of ultrasound‐guided cyst aspiration. Three patients had cyst aspiration more than once. Follow‐up data were available for 29/39 (74%) patients who had successful ultrasound‐guided cyst aspiration and received a subsequent ultrasound assessment in our clinic. In 8/29 (28%) patients, the cyst had completely resolved. In total, 4/21 (19%) patients with a persistent cyst opted for elective surgical intervention, while 17/21 (81%) continued conservative management at the last follow‐up. Overall, 25/29 (86%) of those followed up after successful ultrasound‐guided cyst aspiration, and at least 25/46 (54%) of all patients, avoided any form of surgical treatment.

**Conclusions:**

Ultrasound‐guided cyst aspiration is an effective treatment for the management of acute adnexal torsion due to a cyst with unilocular morphology and anechoic or hypoechoic fluid content. With the use of this technique, emergency hospital admission and surgery were avoided in the majority of patients. © 2025 The Author(s). *Ultrasound in Obstetrics & Gynecology* published by John Wiley & Sons Ltd on behalf of International Society of Ultrasound in Obstetrics and Gynecology.

## INTRODUCTION

Adnexal torsion is a gynecological emergency in which the ovary and/or Fallopian tube twist on their suspensory ligaments, leading to compromise of their vascular supply. If the structures remain in this position, hypoxia leads to necrosis and permanent loss of tissue function. Adnexal torsion commonly involves both the ovary and the Fallopian tube, but it can also involve either structure in isolation. Adnexal torsion often occurs in the context of a cyst, most commonly functional ovarian cysts, benign cystadenomas or dermoid cysts^1^.

Historically, the management of adnexal torsion was with emergency open or laparoscopic salpingo‐oophorectomy, with the objective of treating acute pain and preventing potential complications such as peritonitis. In recent years, multiple studies have shown that adnexal structures that appear to be necrotic at initial surgical intervention, if preserved, are able to regain function after they are detorted^2^, ^3^, ^4^. As such, emergency surgical treatment for adnexal torsion has become more conservative, involving detorsion of the affected ovary and/or Fallopian tube^5^, which can usually be achieved laparoscopically^6^. If the cyst is functional in nature, no further treatment is necessary. Non‐functional cysts may require secondary elective surgery, either because they cause symptoms or in order to reduce the risk of recurrent torsion.

Ultrasound‐guided cyst aspiration may be an alternative management option for adnexal torsion in which a simple cyst is present. This can be carried out under local anesthetic in the outpatient department via a transabdominal or transvaginal route. The cyst is aspirated in its entirety, which relieves the torting force (torque) applied to the pedicle and facilitates detorsion.

In the literature to date, a number of case reports and small case series have reported the use of ultrasound‐ guided cyst aspiration for the management of adnexal torsion^7^, ^8^, ^9^, ^10^, ^11^, particularly among patients undergoing assisted conception^7^, ^8^, ^11^. However, there are no previous reports of this technique being offered in a systematic manner to consecutive suitable acute gynecology patients. The objective of this study was to review all cases of ultrasound‐guided cyst aspiration carried out in our center for the management of adnexal torsion and report its efficacy.

## METHODS

This was a single‐center study of patients who underwent ultrasound‐guided cyst aspiration in the outpatient setting for the management of adnexal torsion. Cases were identified retrospectively from all patients who attended the Gynaecology Diagnostic and Outpatient Treatment Unit at University College London Hospital (UCLH), London, UK, between February 2008 and September 2023. We sought advice from the National Health Service Health Research Authority and the Joint Research Office at UCLH regarding ethical approval. We were advised that formal ethics approval was not needed for this study, as the data had already been collected as part of routine care and anonymized and analyzed within the care team. This study was registered with the Research Registry with the unique identifying number researchregistry10425.

All patients referred to our center with symptoms suggestive of adnexal torsion are offered a transvaginal and/or transabdominal ultrasound scan using high‐resolution equipment (Voluson 730 and E8 Expert; GE Healthcare, Zipf, Austria). Ultrasound scans are performed by experienced (Level‐II)^12^ ultrasound operators. A diagnosis of adnexal torsion is made by taking into account both clinical and ultrasonographic features. The findings of the initial ultrasound examination, clinical diagnosis of adnexal torsion and suitability of ultrasound‐guided cyst aspiration are confirmed by Level‐III^12^ ultrasound operators who are consultant gynecologists with expertise in gynecological ultrasound. A positive ultrasound diagnosis of adnexal torsion is made in the presence of at least one of the following criteria: ovarian edema, the ‘whirlpool sign’ (the ultrasound appearance of a twisted adnexal pedicle) or the affected ovary being located anterior to the uterus in the uterovesical space^1^. In women with no specific ultrasound features of adnexal torsion, the diagnosis of torsion is made clinically, based on the patient's history of acute pelvic pain ipsilateral to a cystic lesion, with no evidence of other abnormalities that could explain the presenting symptoms.

Patient selection and aspiration procedures took place according to the parameters and methods outlined in detail below. Success was defined as the avoidance of emergency surgery for adnexal torsion related to the same cyst. Emergency surgery was defined as an operation that required immediate admission to hospital to prevent organ damage, permanent disability or death^13^.

### Patient selection

Suitability for safe ultrasound‐guided cyst aspiration under local anesthetic was assessed using the following principles. First, the nature of the adnexal cysts was determined using the pattern recognition method, for which our unit has an accuracy of 97% (sensitivity, 94%; specificity, 96%; area under the receiver‐operating‐characteristics curve, 0.95) for discriminating between benign and malignant lesions in the outpatient setting^14^. In order to be eligible for aspiration, the cyst must appear benign with no features suggestive of borderline or malignant potential. Generally, such cysts will be thin‐walled and unilocular, and demonstrate no or minimal vascularity on color‐Doppler examination. Second, the cyst must be amenable to aspiration. This means that it must be unilocular (or have a low number of locules) and have fluid content, thus excluding dermoid cysts. Third, the cyst must be accessible for aspiration via either a transvaginal or a transabdominal route. This means that it must be possible to apply the ultrasound probe close to the cyst wall with avoidance of the urinary bladder, bowel and large blood vessels. Finally, the patient must opt for ultrasound‐guided cyst aspiration, having undergone an informed consent process during which they are informed that emergency surgery under general anesthetic is the standard management for acute adnexal torsion and are offered the alternative of proceeding directly to surgery. Patients who present with adnexal torsion in the absence of a cyst, or for whom aspiration is not suitable (for example, those with a dermoid cyst), are offered routine treatment with emergency surgical management under general anesthetic.

**Figure 1:**
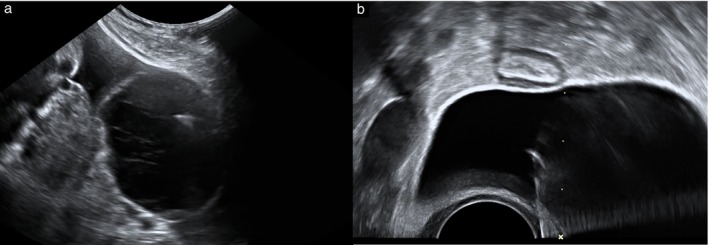
Grayscale ultrasound images showing technique for ultrasound‐guided cyst aspiration via transabdominal (a) and transvaginal (b) route in patients with acute adnexal torsion.

The choice between the transvaginal and transabdominal approaches is predominantly guided by the location of the cyst and the route allowing the most direct safe entry. If both options are technically equivalent, a transabdominal route is generally preferred as a sterile field can be maintained, minimizing the risk of infection. A transvaginal approach is not usually offered if patients have never been sexually active. The techniques for ultrasound‐guided cyst aspiration via the transabdominal and transvaginal routes are described herein and illustrated in Figure 1.

### Transabdominal cyst aspiration

When using the transabdominal route, the patient is positioned supine. The abdominal skin is cleaned with an antiseptic solution containing 4% chlorhexidine in isopropyl alcohol (Hibiscrub®; BCM Ltd, Nottingham, UK). Sterile drapes are applied to the skin around the area overlying the cyst. The abdominal ultrasound probe is prepared for use in the standard manner using a disinfectant wipe and sterile gel. Under ultrasound guidance, the operator infiltrates local anesthetic (10 mL 1% lidocaine) to the abdominal wall overlying the cyst, including the parietal and visceral peritoneum. Once the skin has been anesthetized, the operator uses a freehand technique to introduce a single‐lumen needle with a Luer locking mechanism for syringe attachment, such as an 18‐gauge spinal needle (Becton Dickinson & Co., Franklin Lakes, NJ, USA), through the abdominal skin and into the cyst wall under ultrasound guidance. Once the tip of the needle has been confirmed to be correctly sited within the cyst, the stylet is removed and the cyst content is aspirated. Once the cyst has been emptied completely, the operator withdraws the needle. Direct ultrasound guidance is maintained throughout.

### Transvaginal cyst aspiration

When using the transvaginal route, the patient is positioned in the lithotomy position and sterile drapes are applied. The vulva and vagina are cleaned with an antiseptic solution such as Prontosan® Wound Irrigation Solution (B. Braun Medical Ltd, Sheffield, UK). The vaginal ultrasound probe is prepared for use in the standard manner using a disinfectant wipe, a sterile probe cover and sterile gel. A disposable endocavity needle guide (CIVCO Medical Solutions, Kalona, IA, USA) is fixed to the vaginal ultrasound probe. The operator inserts the probe, which is positioned so that the probe and needle guide directly oppose the vaginal wall overlying the cyst. A single‐lumen needle with a Luer locking mechanism for syringe attachment, such as an 18‐gauge 300 mm aspiration needle (Vitrolife, Västra Frölunda, Sweden), is used to both instill local anesthetic and aspirate the cyst. Prior to aspiration, the vaginal wall is infiltrated with local anesthetic (10 mL 1% lidocaine) under ultrasound guidance. Once the vaginal wall has been anesthetized, the operator advances the needle so that local anesthetic can be instilled within the parietal and visceral peritoneum. After the administration of local anesthetic, the needle is advanced into the cyst lumen. An empty 20‐mL syringe is attached to the needle and the cyst content is aspirated. Once the cyst is completely empty, the aspiration needle is withdrawn from the needle guide, following which the ultrasound probe is removed from the vagina.

### Follow‐up

A sample of cyst content is routinely sent for cytological examination. A single dose of prophylactic antibiotic is administered to the patient, such as 625‐mg co‐amoxiclav orally or a suitable alternative in case of penicillin allergy. Patients are monitored in the clinic to assess for resolution of their pain. Those who experience persistent or recurrent symptoms after ultrasound‐guided cyst aspiration are admitted for emergency surgical management of adnexal torsion under general anesthetic. Those whose symptoms have resolved are discharged. Patients are informed about the risk of recurrence of adnexal torsion and the need to seek urgent medical attention if their pain recurs. A follow‐up ultrasound scan is organized, usually 6 weeks following the procedure, with further follow‐up determined by the findings and patient preferences. In pregnant patients, the timing of follow‐up may be adjusted to offer earlier reassurance about the health of the pregnancy or to fit with their routine obstetric care. Those with recurrent cysts in pregnancy are offered follow‐up at 3 months postpartum so that further management can be discussed.

### Statistical analysis

Patient demographic and clinical data and ultrasound images and data were stored in our clinical database (Viewpoint Version 5; Bildverargeritung GmbH, Munich, Germany). This includes background clinical data, scan findings, procedural details and clinical outcomes.

Data analysis was performed using GraphPad Prism v10 (academic license) (GraphPad Software, San Diego, CA, USA). Non‐parametric variables are expressed as median and interquartile range (IQR). The variables analyzed included age, parity, pregnancy status, previous abdominal surgery, presence of ovarian edema on ultrasound, anterior location of the torted ovary and route of aspiration (transabdominal or transvaginal). Categorical values were binary coded for analysis (as 1: transabdominal aspiration route, parous, previous abdominal operation, previous diagnosis of endometriosis, ovarian cyst location, present ovarian edema and anterior displacement of the ovary). Odds ratios, 95% CIs and *Z*‐scores were calculated. Multiple logistic regression analysis was performed to identify demographic, procedural and clinical characteristics associated with the success of outpatient ultrasound‐guided cyst aspiration. Multicollinearity and parameter covariance were also assessed to review variable redundancy. *P* < 0.05 was considered to indicate statistical significance.

## RESULTS

**Figure 2:**
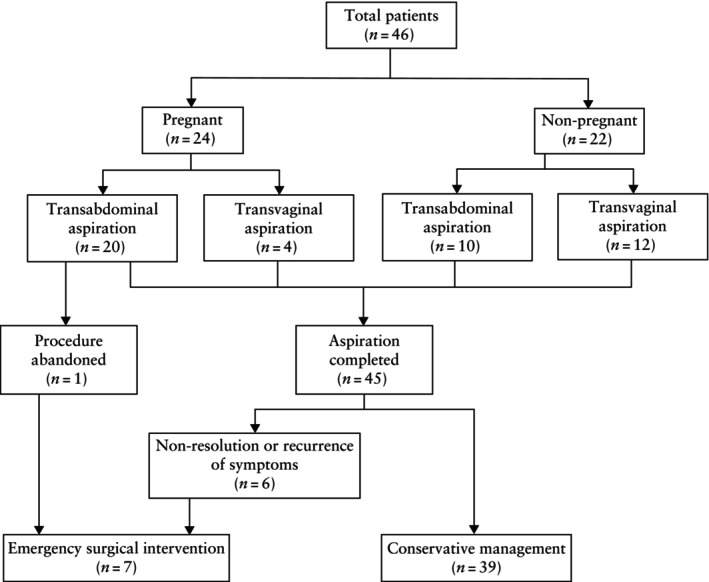
Flowchart summarizing outcomes of patients who underwent ultrasound‐guided cyst aspiration for management of acute adnexal torsion.

During the study period, a total of 46 patients underwent ultrasound‐guided cyst aspiration for the management of adnexal torsion. A flowchart showing patient outcome is presented in Figure 2. Three patients had multiple procedures: two patients had two aspirations and one patient had three aspirations.

### Patient characteristics

All 46 patients who underwent ultrasound‐guided cyst aspiration for adnexal torsion were premenopausal and 24 (52%) were pregnant. Of those who had ultrasound‐guided cyst aspiration while pregnant, 15/24 (63%) were under 12 weeks' gestation, 7/24 (29%) were between 12 and 23 weeks and 2/24 (8%) were between 24 and 28 weeks. Patient demographic and clinical characteristics are displayed in Table 1 and their symptoms at presentation are displayed in Table 2.

**Table 1:** Demographic and clinical characteristics of patients who underwent ultrasound‐guided cyst aspiration for management of acute adnexal torsion (*n* = 46)

Characteristic	Value
Age at presentation (years)	28 (24–33)
Parity	
0	32 (70)
1	10 (22)
≥ 2	4 (9)
Gravidity	
0	18 (39)
1	10 (22)
≥ 2	18 (39)
Gynecological abnormality	
None	34 (74)
Fibroids	4 (9)
Polycystic ovarian morphology	4 (9)
Endometriosis	1 (2)
Adenomyosis	1 (2)
Cervical intraepithelial neoplasia	1 (2)
Congenital uterine anomaly (uterus didelphys)	1 (2)
Surgical history	
None	25 (54)
Surgical termination of pregnancy	6 (13)
Cesarean section	4 (9)
Surgical management of miscarriage	2 (4)
Laparoscopic ovarian cystectomy and appendectomy	2 (4)
Hysteroscopy and laparoscopy for assessment of tubal patency	1 (2)
Laparoscopic ovarian cystectomy and Cesarean section	1 (2)
Laparoscopic ovarian cystectomy and LLETZ	1 (2)
Laparoscopic ovarian cystectomy and hysteroscopy	1 (2)
Laparoscopy for treatment of endometriosis and Cesarean section	1 (2)
Appendectomy	1 (2)
Cesarean section and appendectomy	1 (2)
Hormonal contraception	
None	33 (72)
Levonorgestrel IUD	5 (11)
Copper IUD	2 (4)
Combined hormonal contraception	5 (11)
Medroxyprogesterone acetate injection	1 (2)

Data are given as median (interquartile range) or *n* (%).

IUD, intrauterine device; LLETZ, large loop excision of transformation zone.

**Table 2:** Symptoms at presentation of patients who underwent ultrasound‐guided cyst aspiration for management of acute adnexal torsion (*n* = 46)

Symptom	Value
Acute abdominal pain	25 (54)
Acute abdominal pain + vomiting	19 (41)
Acute abdominal pain + diarrhea + difficulty passing urine	1 (2)
Chronic abdominal pain*	1 (2)

Data are given as *n* (%).

* Pain reported to have been ongoing intermittently for 9 months.

### Ultrasound characteristics

All adnexal cysts were classified, using the pattern recognition method, as benign. The morphological characteristics of the cysts are shown in Table 3 and the ultrasound features of adnexal torsion are shown in Table 4.

**Table 3:** Cyst morphology in patients who underwent ultrasound‐guided cyst aspiration for management of acute adnexal torsion (*n* = 46)

Characteristic	Value
Location	
Ovarian	41 (89)
Paraovarian	4 (9)
Ovarian + paraovarian	1 (2)
Echogenicity	
Anechoic	44 (96)
Low‐level echogenicity	2 (4)
Internal structure	
Unilocular	46 (100)
Presumed histological diagnosis	
Simple functional cyst	42 (91)
Benign epithelial cyst	4 (9)
Cytology	
Benign cyst content, no epithelial cells identified	37 (80)
Benign cyst content, epithelial cells identified	3 (7)
Inadequate for cytological examination	6 (13)

Data are given as *n* (%).

### Procedure characteristics

All ultrasound‐guided cyst aspirations were carried out by consultant gynecologists who were Level‐III ultrasound operators^12^. The transabdominal route was used in 30/46 (65%) and the transvaginal route was used in 16/46 (35%) of patients. In the subgroup of pregnant patients, transabdominal cyst aspiration was carried out in 13/15 (87%) under 12 weeks' gestation and in 7/9 (78%) between 12 and 28 weeks. This difference was not statistically significant (*Z* = 0.31917; *P* = 0.37). The median volume of aspirated fluid was 125 (IQR, 55–200) mL.

### Efficacy

In total, 39 of the patients (85%) experienced complete resolution of symptoms immediately after ultrasound‐guided cyst aspiration. The remaining seven (15%) patients underwent laparoscopic management of adnexal torsion. One patient complained of severe discomfort during aspiration, which was therefore abandoned after 40 mL of cyst content had been aspirated. Six patients reported improvement of symptoms immediately after aspiration but their pain recurred within 1–13 days, necessitating emergency laparoscopic surgery. Of these, two patients had recurrence of pain within hours after surgery. The other four patients presented with recurrence of pain after 2 days, 3 days, 5 days or 13 days. In all 6 patients, laparoscopy was performed and the ovary was preserved. However, the patient who presented 13 days after ultrasound‐guided cyst aspiration had an emergency salpingectomy for a necrotic Fallopian tube. This patient initially presented with a 1‐week history of unilateral abdominal pain prior to ultrasound‐guided cyst aspiration for a torted paraovarian cyst. Although she experienced resolution of pain after the procedure, she developed pyrexia and was treated for a presumed infection. In view of her subsequent re‐presentation and the operative findings, it is likely that necrosis of the Fallopian tube, rather than infection, was responsible for her pyrexia.

Ultrasound‐guided cyst aspiration was successful in 22/24 (92%) pregnant patients and 17/22 (77%) non‐pregnant patients. The procedure was successful in 28/30 (93%) patients in whom it was carried out transabdominally and 11/16 (69%) patients in whom it was carried out transvaginally. Using multiple regression analysis, no statistically significant associations between the variables tested and success of outpatient ultrasound‐guided cyst aspiration were identified.

### Safety

No complications such as hemorrhage or organ injury occurred after ultrasound‐guided cyst aspiration. Cytological analysis of cyst content did not identify malignant cells in any of the aspirated cysts. Of the seven cases in which emergency surgery was carried out, histopathology reports were available for two patients, since most patients underwent laparoscopic detorsion only. In one case, histopathology demonstrated a benign mucinous cystadenoma. In the other, it showed an infarcted benign cyst and partially infarcted Fallopian tube with no evidence of serous tubal intraepithelial carcinoma or malignancy.

### Pregnancy outcomes

Pregnancy outcome data were available for 15/24 (63%) patients. Nine patients went on to deliver at a different hospital and their outcomes are not captured in this analysis. Of the 15 patients whose delivery occurred at our center, 14 had live births. Of the 14 live births, five had a vaginal delivery and nine delivered by Cesarean section. Of those who had a Cesarean delivery, one patient's cyst persisted and they had a right ovarian cystectomy at the Cesarean section. Histopathology confirmed a benign cyst. In all other patients who had a Cesarean section, the cyst had resolved by the time of delivery. One patient who presented at our center experienced a late miscarriage. The patient had ultrasound‐guided cyst aspiration at 7 + 6 weeks' gestation. Following this, the pregnancy appeared normal on the routine 12‐week ultrasound scan. She presented with heavy vaginal bleeding at 20 weeks and was diagnosed with a spontaneous miscarriage.

### Repeat aspirations

A total of 3/46 (7%) patients had repeat aspirations. A non‐pregnant patient underwent ultrasound‐guided cyst aspiration twice, during two separate presentations, 4 months apart. The patient had been reviewed in the interim period, at which point the cyst responsible for the initial adnexal torsion had resolved. Therefore, the second aspiration was to treat a new functional cyst that had caused recurrent adnexal torsion.

Two pregnant patients had repeat aspirations during the same pregnancy. Both had initial ultrasound‐guided cyst aspiration for adnexal torsion of a simple cyst between 10 and 12 weeks' gestation. They were reviewed 1–2 weeks later, at which point they were asymptomatic, but re‐presented with recurrence of pain thereafter. At their repeat presentations they were offered laparoscopic detorsion under general anesthetic, but declined and underwent a second ultrasound‐guided cyst aspiration. One of the two patients presented a third time and again declined surgery, and therefore had a total of three ultrasound‐guided cyst aspirations. This patient was followed up at 3 months postpartum, at which point the ovarian cyst had resolved and both ovaries had a normal sonographic appearance. Both of the other patients who had repeat aspirations also had complete resolution of the cyst at follow‐up.

**Table 4:** Ultrasound features of adnexal torsion in patients who underwent ultrasound‐guided cyst aspiration for management of acute adnexal torsion (*n* = 46)

Feature	Value
None	21 (46)
Ovarian stromal edema	13 (28)
Ovarian stromal edema + ovary located anterior to uterus	5 (11)
Ovary located anterior to uterus	4 (9)
Ovarian stromal edema + edema of ipsilateral Fallopian tube	1 (2)
Ovarian stromal edema + ovary located anterior to uterus + edema of ipsilateral Fallopian tube	1 (2)
Ovarian stromal edema + whirlpool sign	1 (2)

Data are given as *n* (%).

### Follow‐up data

Follow‐up data were available for 29/39 (74%) patients who did not require emergency surgery. The median time until follow‐up ultrasound assessment was 28 (IQR, 9–42) days. Complete cyst resolution was diagnosed in 8/29 (28%) cases. Four of 21 (19%) patients with a persistent cyst opted for elective laparoscopic surgery while 17/21 (81%) opted for conservative management. As such, at least 25/46 (54%) patients who had ultrasound‐guided cyst aspiration for acute adnexal torsion at least once avoided abdominal surgery altogether. The remaining 10 patients either did not attend follow‐up or were planned for follow‐up at their local hospital; therefore, their data were not available for this analysis.

## DISCUSSION

This study demonstrates that ultrasound‐guided cyst aspiration is an effective treatment for acute adnexal torsion caused by a unilocular cyst with anechoic or hypoechoic fluid content, and it allowed emergency surgery to be avoided in 85% of patients. All reported procedures took place in the outpatient department and no complications occurred as a result of ultrasound‐guided cyst aspiration. Of the seven patients in whom ultrasound‐guided cyst aspiration was ineffective and who required emergency laparoscopy, none had an oophorectomy.

The avoidance of emergency surgery has clear benefits for all patients, including immediate relief of pain and avoidance of hospital admission, surgical risks and anesthetic risks. It is of particular benefit to pregnant patients, who can defer surgery until after delivery, especially those at advanced gestations, when laparoscopic surgery cannot be performed safely and open surgery may be required. All pregnant patients in this series were under 28 weeks' gestation. However, we believe the technique could also be performed in patients at advanced gestation and could be used to facilitate vaginal birth in cases of low‐lying cyst that obstructs labor. Avoiding emergency surgery is also especially beneficial for patients with a complex surgical history or medical comorbidities that increase their personal risk of surgical or anesthetic complications. Ultrasound‐guided cyst aspiration avoids surgical handling of ovarian tissue when it is friable, edematous and more likely to bleed, which may require surgical repair that can compromise ovarian reserve, or even necessitate oophorectomy. Indeed, although conservative surgery to detort the ovary is recommended in modern practice, some studies report rates of oophorectomy at laparoscopy for adnexal torsion to be as high as 50–70%^15^, ^16^, ^17^. Furthermore, ultrasound‐guided cyst aspiration is particularly advantageous for patients with adnexal torsion secondary to functional cysts, who are unlikely to require any subsequent elective surgery as functional cysts will not recur after aspiration. This was the case for just under one‐third of those followed up in this study.

Our experience is that the procedure of ultrasound‐guided cyst aspiration is well tolerated, with most patients reporting minimal discomfort after the infiltration of local anesthetic. Indeed, only a single aspiration was terminated prior to completion owing to patient discomfort, and several patients opted for repeat aspirations, even when surgical intervention was recommended to them. Prospective studies with a focus on patient experience would be of value to formally assess procedure‐related pain and tolerability.

It is reassuring that no complications were encountered after a total of 50 procedures were performed in 46 patients. The main safety considerations are visceral injury, inadvertent puncture of a malignant cyst, intra‐abdominal bleeding and infection. The rates of visceral injury in a procedure conducted under continuous ultrasound guidance by a highly trained operator are likely to be very low. Therefore, a much larger study would be required to statistically define such risks. Although such studies do not exist for patients with adnexal torsion, there is extensive experience of ultrasound‐guided follicle aspiration for oocyte retrieval in *in‐vitro* fertilization (IVF). This similarly involves the introduction of a needle to the pelvis via a transvaginal or transabdominal route under ultrasound guidance, and has been shown to be overwhelmingly safe^18^. Ultrasound‐guided cyst aspiration for adnexal torsion is more straightforward and less traumatic than oocyte retrieval, so it is likely to have even lower complication rates. Prior to the introduction of transvaginal ultrasound, the transvesical route was used routinely for transabdominal follicle aspiration for IVF and it has also been described for other invasive gynecological procedures^19^, ^20^. Although transvesical puncture is safe, it may be more uncomfortable for patients and carries a small risk of severe hematuria. In view of this, we have avoided using a transvesical approach to date. However, we would consider carrying out transvesical cyst aspiration if it was impossible to bypass the bladder while performing cyst puncture transabdominally.

One pregnant patient in this series experienced a second‐trimester pregnancy loss after cyst aspiration performed at 7 + 6 weeks. Following this, the pregnancy appeared normal on the routine 12‐week scan. In view of this, it is very unlikely that the pregnancy loss was related to the cyst aspiration.

This is the largest study of ultrasound‐guided cyst aspiration to date and the first to describe regular use of the technique as an alternative to emergency laparoscopy in consecutive patients presenting with acute adnexal torsion. This represents a novel approach to the management of torted adnexal cysts. In our experience, the technique can be used for both ovarian torsion and isolated tubal torsion secondary to paraovarian cysts^21^.

The main limitation of this study is its retrospective nature, which means that data for patients who did not undergo ultrasound‐guided cyst aspiration were not collected. This lack of a denominator means that we are unable to report the proportion of cases of adnexal torsion that can be treated with ultrasound‐guided cyst aspiration. Being retrospective, data on long‐term outcomes are incomplete owing to a lack of planned long‐term follow‐up.

We recognize that this procedure requires immediate access to skilled ultrasound operators, who are not available in all healthcare settings. This is vital to ensure that the cyst is accurately characterized as benign in order to facilitate safe aspiration. Adnexal torsion is a clinical diagnosis and expedient detorsion is critical to preserve long‐term tissue function, so consideration of ultrasound‐guided cyst aspiration should not delay intervention when torsion is suspected and expertise for ultrasound‐guided cyst aspiration is not immediately available. However, it does provide a timely and straightforward treatment option when it can be facilitated. The development of competence to carry out this procedure should be considered by gynecologists and reproductive medicine specialists already skilled in ultrasound, who are well placed to offer this effective and less invasive treatment option.

In conclusion, ultrasound‐guided cyst aspiration is an effective and less invasive treatment for acute adnexal torsion associated with unilocular cysts with anechoic or hypoechoic fluid content. The procedure allows the majority of patients to avoid the risks associated with emergency laparoscopic surgery and general anesthetic, as well as avoiding hospital admission and a surgical recovery period. It confers particular benefit on pregnant patients and those for whom surgical or anesthetic risks are increased, as well as those with functional cysts who are unlikely to need any subsequent elective surgery.

## Data Availability

Data sharing is not applicable to this article as no new data were created or analyzed in this study.
